# Designing an eSports intervention for middle-aged and older adults in Hong Kong: Social marketing approach

**DOI:** 10.1371/journal.pone.0284504

**Published:** 2023-04-27

**Authors:** Ka-Man Leung, William Chu

**Affiliations:** Department of Health and Physical Education, Faculty of Liberal Arts and Social Science, The Education University of Hong Kong, Hong Kong, China; Universiti Malaya, MALAYSIA

## Abstract

This study examined the perceptions and experiences of middle-aged and older adult participants in electronic sports (eSports) in Hong Kong (HK), China, by using the social marketing (SM) approach. This qualitative study applied SM approach to the design of a center-based eSports intervention for middle-aged and older adults in HK. Interviews were conducted with 39 adults stratified in terms of age (i.e., 45–64 vs. ≥65 years) and experience with eSports. Ten administrators working in community elderly centers were invited for semistructured interviews. Thematic analysis was performed on the data by incorporating SM. Main findings are presented in terms of five P’s. The *product* component of an eSports intervention includes the foundation of eSports (e.g., safety, eSports training), suitable games for older adults, and professional equipment (e.g., large-screen devices and motion-controlled Nintendo Switches). The *price* component comprises affordability and the frequency and duration of each eSport session, and the *place* component includes accessibility and spaces to play eSports. The *promotion* component should be educational in nature and can incorporate free trials and gaming days, short films about older adults playing eSports, promotional channels, physical evidence and annual eSports competitions. The *people* component consists of support from administrators and the center in charge, the availability of skilled program instructors and staff, and appropriate partnering, team sizes and instructor-to-participant ratios. The 5P’s enhance the design of future center-based eSports interventions and can help researchers and practitioners determine which aspects encourage middle-aged and older adults to participate in eSports.

## Introduction

### Population aging and active aging

Global population aging has created serval public health challenges, such as the lack of long-term care facilities and adaptive environments for older adults [1]. Projections indicate that Hong Kong (HK) will have one older adult (aged 65 years or older) for every three people by 2036 [2]. As a result, caring for older adults and active aging have been key government policy objectives in HK, with related recurrent expenditures accounting for an estimated HK$91.9 billion (20.8% of the total government expenditure) from 2019 to 2020 [3]. In HK, the District Elderly Community Centre (DECC) and Neighborhood Elderly Centre (NEC) provide community support at the district and neighborhood levels, which enables older adults to lead healthy and dignified lives. Their targeted service groups include older adults aged 60 years or older, their caregivers, and the community at large. The services include social, recreational (physical activity), and developmental activities, and health education [4]. Both the DECC and NEC play key roles in active aging and aging in place.

### Electronic sports (eSports) and older adults

To meet the needs of an aging population, the Hong Kong Special Administration Region (HKSAR) Government has promoted gerontechnology, which combines gerontology and technology. The Gerontechnology Platform, an initiative funded through the HKSAR Government’s Social Innovation and Entrepreneurship Development Fund, leads collaborative efforts within the gerontechnology ecosystem. In 2017, Our Hong Kong Foundation overviewed the HK gerontechnology industry and revealed that an insufficient understanding of older adults’ perceptions of and demand for technology have resulted in certain gerontechnologies not being accessible (e.g., eSports). In addition, a lack of research on gerontechnology has been reported [5]. Academic studies have revealed that older adults in HK have a positive attitude toward technology (e.g., [6, 7]) and that they mainly use technology for entertainment (e.g., video games) and communication [7].

Video games are a form of interactive digital entertainment on computers, mobile phones, tablets, or consoles. Video games with a competitive element are collectively known as eSports. In 2018, Acer and Senior Citizen Home Safety Association held an eSports activity for older adults in HK that benefitted the participants’ health and strengthened their relationships with their families [8]. Although some studies have revealed associations between eSports and adverse health outcomes such as sleep disturbance [9, 10], overuse strain [10], obesity [11], behavioral problems [12], sedentary behavior [13], and high levels of stress [14], others [10, 15] have indicated that eSports can benefit physical [10, 16], psychological [10, 17], and social health [10, 18]. Leung and her team also did a pilot study to examine the impact of an eight-week eSports intervention (using Fitness Boxing, Nintendo Switch) to improve the psychosocial health, physical and cognitive functions of 54 healthy older adults in 2021. Preliminary results revealed that the participants in the eSports group had better performance in the tests of agility and balance, cognitive ability, higher upper and lower limbs flexibility than those in the control group. There is a clear research gap in related literature that more understanding about eSports ecosystem such as eSports participation and its health impact is warranted [15]. One approach to analyzing eSports ecosystem is to explore the perceptions and experiences of participants and administrators. Few studies have investigated eSports interventions in community elderly centers that promote active aging among middle-aged and older adults in HK.

### Theoretical framework

The theory of planned behavior (TPB; [19]), the Social Ecological Model (SEM) [20], Social Cognitive Theory (SCT; [21]), and Social Marketing (SM; [22]) have been widely used to analyze behavior. For example, Netz and Raviv [23] explored differences in motivation to engage in physical activity (PA) among age groups through SCT. Using the SEM, Vance et al. [24] studied the effects of PA and sedentary behavior on cognitive health in older adults. Using the TPB, Leung and Chu. [10] identified the behavioral, normative, and control beliefs of middle-aged and older adults in HK participating in eSports.

SM is based on other bodies of knowledge (e.g., marketing and communication theory) and is used to determine how the behavior of target populations can be changed [25]. Numerous scholars have used SM to study alcohol addiction [26] and encourage PA among adults aged 60 years and older [27]. “SM is the adaptation of commercial marketing technologies to programs designed to influence the voluntary behavior of target audiences to improve their personal welfare and that of the society” (p. 348) [22]. SM involves using segmentation to identify homogenous groups (e.g., inactive older adults) from a heterogenous population (e.g., older adults), address the needs of consumers [28, 29], and trigger behavioral change (e.g., encouraging individuals to play eSports; [30]). SM incorporates the concepts of product, price, place, and promotion from traditional marketing; these are known as the four P’s [29]. Products are the tangible and intangible results (e.g., eSports interventions) of an exchange. Price refers to the monetary and nonmonetary factors (e.g., time, effort, and energy) required of a behavior [31]. Place refers to the locations of the target segment and product when the target segment engages in behavioral change. Promotion refers to advertising initiatives that raise awareness and convey a particular message [27]. Booms and Bitner [32] introduced three additional factors, namely physical evidence, people, and processes. The people component refers to those directly and indirectly involved in the design, marketing, and sale of a product or service, including management teams and customers.

SM enables social marketers (administrators, in this study) to learn about target segments and design effective interventions (eSports, in this study) to meet target segments’ needs (middle-aged and older adults, in this study). The more fully the SM approach has been adopted in the planning and provision of a behavioral change activity, the more successful the target segment’s adoption of the behavioral change [28]. This study added the people component to the 4 P’s to identify factors critical to the success of an intervention and the effects of incorporating SM into the intervention [33].

### Objectives

This qualitative study used SM to design an eSports intervention for community or elderly centers in HK China. It is envisaged that the study results will i) enlighten the research in design parameters of a successful intervention to promote center-based eSports programs for the benefits of the aforementioned studied population, and ii) facilitate long-term healthy and active aging.

## Materials and methods

### Participants

The intervention was implemented in the DECC and NEC to access the target segment, namely middle-aged and older adults. Participants were required to be aged 45 years or older and able to communicate in Cantonese. The participants comprised 39 adults aged 45 years or older. The participants were stratified in terms of age (i.e., 45–64 vs. ≥65 years) and experience with eSports s. Ten administrators working in the DECC and NEC also participated.

### Procedure

A cover letter explaining the study was sent to the DECC and NEC. After written consent from the center in-charge (CIC) was received, participants were recruited using advertisements at the target centers. Participants were provided additional information regarding the study (e.g., the definition of eSports) over the phone or by the CIC. The CIC contacted those who agreed to participate and invited them to semistructured interviews at a convenient time. Before each interview, the participants completed a written consent form and information sheet that explained the purpose of the study, the role of the participants, and the potential risks and benefits of participation. All research activities were reviewed and approved by the University Research Ethics Committee (approval number: 2019-2020-0300).

### Semistructured interviews

All interviews were conducted in Cantonese by the principal investigator (woman) and a research assistant (man), who were both trained in qualitative research methods, including interviewing. The interviews were based on a standard set of questions used in our previous eSports-related study [10] that had undergone pilot testing with five middle-aged and older adults. Probing questions were asked to deepen the conversation, extract key information, clarify certain matters, and use time efficiently [34, 35]. For instance, administrators were asked about elements that make eSports interventions suitable for community settings. The interviewees had no prior relationship with the interviewers. All interviews were conducted in the community and elderly centers and were audiotaped for data analysis with consent from the interviewees, who were ensured of the confidentiality of their responses and right to withdraw at any time. Each interview lasted an average of 60 min, and each interviewee received a supermarket voucher worth HK$100 to acknowledge their contributions.

### Data analysis

A research assistant with bachelor degree in translation transcribed the interviews verbatim. Then, two independent coders thematically organized the transcripts. To ensure confidentiality, personal details were removed from the transcripts. The researchers read the transcripts multiple times until distinct themes related to the five P’s emerged. Notes made during the interviews were also used as a source for data triangulation to ensure accuracy. Differences in coding were resolved through discussion with the principal investigator. Data saturation was reached when no new codes or themes were identified. To increase the accuracy of the analysis, two interviewees reviewed the transcripts of their interviews. We used 32-point consolidated criteria for qualitative studies [36] to describe our results.

## Results

We conducted 49 semi-structured interviews with 39 middle-aged and older adults as well as 10 administrators. A total of 60% of the administrators (three and seven administrators from public and private communities and elderly centers, respectively) were men, and 90% had a tertiary education or higher. A total of 15 participants were middle aged, and 24 were older adults (n = 39; 17 from two private centers and 22 from two public centers); approximately 70% were women, and 51% had no experience with eSports. Table 1 profiles the participants, and Table 2 presents the themes of the interviews corresponding to the five P’s. Interviewee codes are explained in the note of Table 2.

**Table 1:** Sociodemographic characteristics of participants (n = 49).

	Participants with eSports experience	Participants without eSports experience	Administrators
**Age**			
**Middle-aged (under 64)**	9	12	10
**Older adult (65 and above)**	10	8	
	19	20	10
**Gender**			
**Female**	16	11	4
**Male**	3	9	6
**Occupation**			
**Full time**	0	1	10
**Search for job**	0	1	0
**Retired**	18	16	0
**Part-time job**	1	2	0
**Education level**			
**Primary**	5	5	0
**Secondary**	7	13	1
**Tertiary**	5	2	7
**Master & above**	0	0	2
**Uneducated**	2	0	0
**Monthly family income (HK$)**			
**Nil**	6	9	This question was not asked
**Below 20000**	9	6	
**20000–30000**	1	1	
**30000–40000**	2	3	
**40000–50000**	1	1	
**Estate/Organization type**			
**Public**	6	7	3
**Private**	7	4	7
**Home Ownership Scheme (HOS)**	6	9	0

**Table 2:** Five P’s of the intervention and SM approach.

5Ps	Attributes
**Product**	Intervention elements *Foundation of eSports* *safety precaution* *eSports training* Game content *motion-based games*, *non-horrible game contents* *multi-generation elements* Professional equipment
**Price**	Affordable fees Frequency and duration
**Place**	Accessible location Space
**Promotion**	Educational in nature eSports free trial playing and fun-days Production of a mini-movie Multiple promotional channels Physical evidence Annual eSports competition
**People**	Support from administrators and center-in-charge Availability of program instructors and staff with the required skills and attitude Appropriate pairing and partnering preference Team size and instructor to participant ratio

Note. The themes are detailed in Section 3, with direct quotes indicated by italics. Interviewee codes are explained as follows: MA2: male administrator number 2; MP1: male participant (with eSports experience) number1; FP12: female participant number 12; FA4: female administrator number 4; MNP8: male participant (without eSports experience) number 8.

### Product

#### Elements of the intervention

MA2 stated that programs should cover the foundation and provide a basic understanding of eSports:

*“The theory part…helps [the participants] understand the basics of the game…the rules for winning and losing…the different characters and their specialties*, *and how to best use each character*. *The practical part teaches them how to…marshal and strategize…an entire plan and play a role to achieve their goals*.*”*

MA5 suggested that programs cover the basic elements of eSports:

*“…The program should cover some basic concepts…and the culture of eSports…for example*, *its history…This might interest participants and…encourage them to try something challenging*.*”*

MA3 recommended that tips for communicating, safety precautions (e.g., warm-up and stretching exercises), and sports psychology be incorporated: *“…We should teach them some tips to communicate……simple warm-up and stretching exercises*, *and sports psychology”*

MP1 also recommended that exercise be included: *“We do stretching exercises to prevent wrist strain…and eye muscle relaxation exercises*.*”*

MA5 suggested that participants receive basic eSports training in fine motor skills: *“Whenever I run a new program*, *I teach [the participants] fine motor skills essential to eSports…to make their fingers more sensitive*.*”*

#### Game characteristics

Interesting and simple motion-based games are preferred by participants to war games and those with violence, blood, killings and horror. Some like games suitable for multi-generations so as to foster relationship and harmony with their family and non-family members.

FP12 preferred motion-based games over war-based games: *“I like motion-based games like car racing*. *I don’t like historical war games where ancient figures fight each other with swords and spears; it’s so boring*.*”*

FP15 preferred racing games over violent games: *“I like exciting games like car or horse racing*, *which is more positive…I don’t like games that have killing…and horror (with ghosts and monsters)*, *because they give me nightmares*.*”*

FP3 preferred games with multigenerational elements suitable for all ages because they foster relationships: *“I like games suitable for all ages*. *I’m a teacher*. *We have to do something to solve the…generation gap…problem*.*”* They added: “…*Playing with young people…generates common interests*, *talking points*, *and harmony*. *For example*, *I happily play with my son and grandchild…We play online even though we live in different cities*.*”*

Though older adults have a strong intention to participate in eSports and exergaming, they lack skill and knowledge to carry out the behavior. With relatively low receptive ability, they found it difficult to perform the routines and to comprehend complicated on-screen interfaces.

FP4 stressed the difficulties of beginners: *“We don’t know how to play Esports…which is extremely difficult for beginners…With my cell-phone*, *I video-tape what our instructor teaches us while taking note of each detailed skill of playing…At home*, *I do revision of my full set of notes……”*

MP6 had difficulty in performing steps in right sequence: *“It would be better if we are given a full set of steps guiding us what to do and how to play*. *Without it*, *we sometimes lose the way after skipping a step……”*

FP12 elaborated what suited older adults: *“…We should choose …easy-to-play games for older adults…Games with complicated on-screen interfaces are difficult to comprehend…”*

MP1 echoed: *“…I felt dizzy and very uncomfortable after playing complex games such as PUBG …I was easily shot dead because of slower movements…”*

#### Professional equipment

Most participants preferred devices with large screens: *“Cellphones are too small for older adults to use*. *As you know*, *our eyesight is not good…It is better to use tablets with bigger screens*, *which are easier for us to use”* (FP7). *“I don’t think that our eyes are adversely affected because we use devices with large screens like computers*. *We also use projectors to make images bigger*.*”* (FP11).

Participants also preferred using professional equipment: *“I think that playing the Nintendo Switch is better than playing on cellphones because it’s three-dimensional…”* (FP12). *“There are requirements for eSports equipment…Your home computer must be operable on an eSports level…as with your keyboard*, *mouse*, *and headphones”* (FP8).

One administrator (FA4) remarked: *“Our center…is already equipped with large screens*, *smart televisions*, *Wi-Fi*, *and Nintendo Switches*. *I think…we are ready to run exergames for our members as long as the venue and staff are available*.*”*

### Price

#### Affordability

One participant (FP1) stated: *“Free products are a must to make eSports popular among older adults*.*”* This thought was echoed by two other participants. MA7 noted: *“Free-to-play game sessions could attract older adults*. *If you’re charged HK$50 per game*, *for example*, *players could get refunds after the game*.*”* FP13 indicated: *“The price would determine whether I play*. *I would play if the price were reasonable*, *maybe HK$10 or HK$20 per session*.*”*

#### Frequency and duration

Older adult’s playing habit (e.g., the time of day they played, the duration of each session, and the number of sessions per week) depended on their lifestyles and daily schedules.

FP1 preferred playing during their leisure time in the afternoons: *“I play during my leisure time for less than 2 h per session…one to two times a week*. *I prefer playing in the afternoon when I’m free*.*”*

FP12 preferred 1-h morning sessions of the same frequency: *“I prefer playing in the morning*. *I do household chores and prepare meals in the afternoon*. *I play for approximately 1 h per day one to two times a week*.*”*

FP6 favored afternoon game sessions for 20 min at the most: *“Playing at night is not suitable for older adults because our eyesight is not good enough…Some don’t wake up early in the morning*, *so playing at noon is better…for at most 20 min per session*. *Beyond that*, *we would be tired*.*”*

### Place

#### Accessibility

Another administrator (MA3) stressed the importance of the accessibility of eSports: *“To account for older adults’ physical conditions*, *we used to hold exergaming sessions in easily accessible community centers and close to Metro stations*.*”*

FP13 stated, *“I prefer playing in the nearby community centers I’m familiar with*.*”*

#### Space

One administrator (FA1) remarked that the availability of a space for exergaming is essential: *“The venue is a major issue*. *Our center organizes a lot of activities for older adults*, *which leaves little space for exergaming*.*”* One participant (FP1) stated that an equipped venue was not always available: *“We don’t have many options in terms of venues*. *We either rent a place or play in a remote community center…We used to play here on the third floor in a room with ten computers*.*”*

### Promotion

#### Educational in nature and governmental sponsorship

MA8 suggested, *“Promotion can take many forms*, *such as intergenerational education programs and annual eSports competitions…The prizes should be attractive*.*”*

MA9 highlighted the importance of education programs and the sponsorship role of the government *“…The government should implement more education programs to promote the idea that exergaming could be instrumental to intergenerational communion…Promotion through television advertisement and governmental sponsorship of eSports venues and equipment is also important*.*”*

FP8 remarked: “… Making a topical mini-movie with the theme that ‘eSports is not the privilege of youngsters’ could help promoting eSports in older adults.”

#### Free eSports trials and gaming days

FA10 recommended eSports gaming days: *“To increase the popularity of eSports among older adults*, *more television and mass media promotion*, *trials*, *and gaming days are indispensable*.*”*

Another participant (FP13) stated, *“Organizing free gaming trials in elderly centers is the first step*.*”*

#### Short films

FP13 also noted the benefits of promotional short films: *“Making a short film can boost older adults’ motivation*.*”*

Others also commented on short films: *“Making a topical short film and providing sponsorship for instructors and equipment are crucial”* (MP1). Another stated, *“For older adults*, *the games must be interesting and simple*. *Making a topical short film about eSports for older adults could also help”* (FP8).

#### Promotional channels

One participant (FP5) stressed the promotional effect of word of mouth: *“Games must be fun enough to generate word of mouth among older adults*. *They might recommend them to their friends and encourage them to join*.*”* Another participant (MNP8) made a similar statement: *“We must explain to older adults the benefits of eSports and encourage them to spread word of mouth*.*”*

FNP4 stated, *“Promotion through television advertisement is useful because older adults love to watch television and these advertisements would inform them and their family of which centers are running eSports programs*. *Community elderly centers should host more exergaming events*.*”*

MNP7 stated, *“Community elderly centers should promote events through posters*, *a monthly magazine*, *instant messaging apps such as WhatsApp*, *and social media such as YouTube*.*”*

#### Physical evidence

MA7 noted the promotional effect of prizes or gifts: *“Prizes or gifts…could attract older adults*.*”* FA1 also noted, “*They feel good when they receive something tangible such as small gifts as encouragement*.*”*

Both MA8 and MNP8 shared the promotional effect of social events: *“Holding annual eSports competitions for older adults would have a promotional effect*.*”*

FP15 enjoyed the atmosphere of competitions held in spacious venues with spectators: *“Our first eSports competition was held in a large hall with an audience*. *It was a really good atmosphere*.*”*

### People

#### Perspectives from administrators and the CIC

Positive perspective from administrators can encourage older adults to engage in eSports; however, the CIC was doubtful of the popularity of eSports among older adults.

One administrator (MA3) explained why they organized eSports programs for older adults: *“I found it meaningful to bring happiness to older adults through eSports*.*”* Another two administrators were motivated to continue holding exergames for older adults: *“I felt that …the program enabled them to experience a modern trend”* (MA2). *“I observed from our previous program that they enjoyed playing*, *which is really important”* (FA4).

An administrator (MA5) explained that although their CIC did not object to the program, the center received no direct funding: *“The risk is…low and it could be easily held indoors…However*, *I receive no direct funding from my center*, *probably because [center management] thinks that eSports is merely a gimmick and is unpopular among older adults*.*”*

#### Skilled program instructors and staff

Two participants mentioned the key role of program instructors: *“Program instructors are so kind and teach us patiently*. *We form good relationships*. *I am very happy to play eSports”* (FP13). *“[A short-film maker and eSports activity instructor] once said*: *‘How can you persuade others if you don’t know anything yourself*?*’ This inspires me…to learn it well…The instructor taught us wholeheartedly…He video-taped the whole process for us to practice at home”* (FP3).

One administrator (FA1) shared that the programs depended on the availability of staff in their center: *“Because our staff is limited*, *we can organize one exergaming session per week at the most*. *During the 2-h session*, *members take turns playing*.*”* One participant (MP1) stated, *“Most importantly*, *we need program instructors and facilities*. *We used to play…with three supporting staff*, *but they left*.*”*

(FA4) agreed that high-quality staff can encourage older adults to participate: *“…Good-quality staff foster strong relationships with older adults and attract them to exergaming programs*.*”*

#### Partnering

FP7 noted that partnering older adults with youngsters improves the program: *“I think pairing [older adults] with youngsters improves our performance ……it’s more fun and……we play more seriously…… feeling that the whole event has improved*.*”* FP13 expressed that they enjoyed playing with peers: *“I enjoyed playing with volunteers from the community center*.*”*

FP15 indicated that grandmother–grandchild pairings would be effective: *“I think that grandmother–grandchild parings would work*. *Some older adults care for their grandchildren at home…and most kids like playing eGames*. *Knowing little about eGames*, *grandparents tend to scold their grandchildren for being addicted to them*. *If older adults are paired with their grandchildren and play eGames*, *the games will become talking points instead of points of contention between them*.*”*

#### Team size and instructor-to-participant ratio

Teams of up to ten with an instructor-to-participant ratio of 1:5 were considered optimal: *“We have two instructors to supervise a group of ten players*. *Most eSports games are five players against five*. *One instructor can adequately supervise a five-player team”* MA5.

FA1 commented that team size was subject to the availability of devices and staff: *“…I think eight to ten participants per group is appropriate…depending on the availability of devices and staff*. *Too large a group (with too few devices and staff) would increase waiting time and reduce [older adults’] interest*.*”*

## Discussion

This study examined the perceptions and experiences of middle-aged and older adults participating in eSports in HK through SM to determine the optimal design of eSports intervention in terms of product, price, place, promotion, and people.

### Product

In this study, the product is active aging through participation in eSports.

#### Elements of intervention

Unlike younger generation, who focus on technical skills and winning, older adults, with their wealth of experience, prefer professional approaches and engaging in new experiences. The administrators recommended providing a basic understanding of eSports (e.g., basic concepts and the culture of eSports), training (e.g., communication and fine motor skills), and the safety of eSports (e.g., warm-up and relaxing stretching exercises). The administrators also noted that these elements would attract older adults and help them adapt to eSports. Perceived barriers, such as a lack of confidence and knowledge about technology, as identified by Vaportzis et al. [37], may emerge during the process.

#### Game characteristics

To most of the participants, violence, gore, killing, and horror were strong deterrents from eSports. Most participants preferred cute motion-based games. As in Blocker et al. [38], the older adults disliked violent games. However, unlike in Khalili-Mahani et al. [39], the participants preferred motion-based games such as car racing. The participants enjoyed some of the games which were suitable for multi-generations and helped them strengthen their relationships with their family members, which is one of the expected benefits [40]. Many participants had difficulty in tackling the complicated on-screen interfaces of complex games in which they were easily shot dead because of slower movements. Some felt dizzy and very uncomfortable after playing complex games. All these suggest a need for the design of appropriate game contents, training and support materials to aid in the use of these systems, and reducing the complexity of game contents for the older adult population. These results indicate that games must be adapted to target groups [41] based on their preferences and competence.

#### Professional equipment

To engage in eSports, the older adults required professional equipment. Because some had poor eyesight, they preferred playing on large screens. They also preferred the Nintendo Switch (Nintendo Headquarter at: 11–1 Hokotate-cho, Kamitoba, Minami-ku, Kyoto 601–8501, Japan) to cellphones, which they found too small. One participant (MP1) reported that their community center lacked the professional equipment required for eSports programs for older adults. One administrator (FA4) remarked that their center was equipped with large screens, smart televisions, Wi-Fi, and Nintendo Switches that they used to hold exergame events. These results are consistent with those of Kalinowski et al. [42], who explored the availability of appropriate equipment, and those of Baert et al. [43] who reported that a lack of equipment can discourage PA. The senior-friendly interface of the Nintendo Switch helped the older adults play and enjoy eSports [44].

### Price

Price in our study referred to the affordability, frequency, and duration of center-based eSports programs.

#### Affordability

McDonald et al. [45] reported that older adults have lower income postretirement and that being eligible for concessionary prices (e.g., for booking venues) encourages postretirement engagement in PA. In this study, 87% of the participants were retired and 77% had either no monthly household income or a monthly household income of less than HK$20,000. Most expected to be able to engage in trial gaming sessions before committing to longer-term gaming and also expected to participate in center-based programs free of charge. The sessions must be affordable, for example, HK$10 to HK$20 per 1-h session or HK$50 per course. This result is consistent with the results of Hardy et al. [46], Buman et al. [47], and Costello et al. [48], who indicated that affordability is crucial.

#### Frequency and duration

Price includes the participation fee and fluctuates depending on the number of sessions, their frequency, and transportation costs to reach the venue. Anokye et al. [49] revealed that participation in PA is more sensitive to travel time costs than to monetary costs. Thus, interventions must be offered at times suitable to target groups [33].

In our study, several participants mentioned time (e.g., the time of the session, the duration of session, and the frequency of sessions). Participants used their time to complete high- priority tasks such as household chores and played exergames in their leisure time. Participants played at different times of day depending on their schedules. Some were unwilling to attend afternoon sessions because they ended after dark. Interventions must accommodate these preferences [50]. In Vazquez et al. [51], video game–based interventions ranged from 6 to 60 sessions for 4 to 20 weeks, with the duration of each session ranging from 15 to 30 min.

Weaver et al. [52] reported that a typical video game session lasts 1 to 2 h. The participants in our study noted that approximately 0.5 to 2 h–sessions once or twice a week were suitable, although no guideline has been established for the optimal times for game-based interventions to improve functional and physical skills [53].

### Place

According to SM, the distribution of products can generate opportunities [54].

#### Accessibility

Most of the participants lived either in or close to the community or elderly centers where the eSports activities were held. They preferred playing games in the nearby centers they were familiar with. In the literature on PA, easily accessible venues near participants’ homes are crucial for engaging older adults in PA [50]. To increase accessibility and account for the physical conditions of the participants (e.g., wheelchair users), the sessions were held in community centers in flat areas near MTR stations. These results are similar to those of Baert et al. [55], who reported that stairs, a lack of space and infrastructure, and outdated infrastructure were barriers to PA for older adults.

#### Space

In our study, both the administrators and participants suggested that holding an eSports intervention in spacious (e.g., in a large hall with spectators) venues would encourage participation. The frequency of eSports activities was limited by the availability of space in the centers, which were often reserved for other activities. Therefore, venues must be large because “a venue that’s too small will make the players feel uncomfortable—no one wants a spectator to infiltrate their personal space during tense moments of play” [56].

### Promotion

Promotion in our study was educational in nature and centered on free-trial eSports sessions, gaming days, short educational films, promotional channels, physical evidence. These aspects create opportunities for the target segment to engage in center-based eSports programs.

#### Educational in nature and governmental sponsorship

Our study revealed that education programs to promote eSports and exergaming (e.g., eSports and exergaming is not the privilege of the younger generation) and governmental sponsorship of eSports venue and equipment could attract older adults participants.

#### Free-trial sessions and gaming days

In our study, eSports gaming days were considered promotional. eSports demonstrations and free-trial gaming booths in the community and elderly centers increased the accessibility of eSports for older adults and encouraged them to participate. This is consistent with the result of Anokye et al. [49], who stated that “participation in PA was negatively associated with money prices per occasion.” (p.1).

#### Short film

A short promotional film on older adults in eSports and intergenerational exergaming could be produced to change the perception that eSports caters only to the younger generation and to frame exergaming as feasible for older adults, thereby achieving widespread educational and promotional effects among the general public.

#### Promotional channels

Yin et al. [12] identified the “research and knowledge gaps to addressing the emerging public health need and how to safely promote eSports for either competition or leisure” (p.486). Our study identified a need to explain the benefits of eSports (e.g., its contribution to physical, psychological, and social health) to older adults to spread word of mouth. Promotion through television advertisements would be effective because older adults tend to enjoy watching television; these advertisements also inform older adults and their family about when and where eSports programs are held. Centers can also employ multiple promotional channels such posters, a monthly magazine, instant messaging apps such as WhatsApp, and social media such as YouTube.

#### Physical evidence

The results indicate promotional effect of physical evidence (e.g., small prizes and gifts) presented to the target participants upon completion of the program. This result is consistent with research [55] reporting that incentives such as supermarket vouchers and gifts can be used to encourage older adults’ engagement in PA. Our study revealed that at the community level, promotion could take the form of annual eSports competitions. The critical nature of competitiveness was demonstrated in Souders et al. [57], in which older adults playing competitive multiplayer video games reported higher enjoyment and increased participation adherence than did those playing cooperative video games. In addition, gaming encourages movement and improves health. The International Olympic Committee [58] has also indicated that eSports embodies the values of the Olympic Games. These social events and the atmosphere of competitions held in spacious venues with spectators are considered to be attractive to older adults.

### People

People in our study referred to support from administrators and the CIC, the availability of skilled program instructors and staff, appropriate partnering, team size, and the instructor-to-participant ratio, which are all central to planning and implementing center-based eSports programs.

#### Support from administrators and the CIC

Li et al. [59] revealed the positive effect of playfulness on older adults. The administrators reported that enjoyment and novel experiences among older adults during eSports are meaningful, facilitate communication between generations, and foster friendship. This positive image is crucial to initiating and marketing eSports interventions for target groups. The CIC advocated for the eSports programs because the risk is low and the intervention could be easily held indoors. However, some CICs hesitated to fund eSports programs because they perceived them to be a gimmick and unpopular among older adults; instead, funds were allocated to more mainstream activities.

#### Skilled program instructors and staff

The shortage of supporting staff limited the frequency of the eSports programs. Supporting staff with the required skills (e.g., establishing necessary facilities and teaching older adults how to play), attitudes (e.g., being amicable), and experience with older adults (e.g., being aware of the physiological needs of older adults, who must rest intermittently during an eSports session) is essential. In our study, the older adults memorized and performed steps and routines in the correct sequence with difficulty. Thus, the availability of a program instructor who trains and inspires participants is essential. In addition, familiarization with new technology is essential for older adults to engage in PA programs [60], and an assistant must be present to help the older adults [61].

#### Partnering

Some participants enjoying playing with younger people, and others preferred playing with peers. For instance, FP7 stated that the partnering of older and younger participants improved the event because the younger people improved their performance. FP15 noted that grandmother–grandchild pairing generated talking points instead of competition. However, FP13 preferred to play with peers, such as volunteers from the community center, perhaps because they had similar experiences and interests. This result suggests a need for further research on the optimal mode of partnering.

#### Team size and instructor-to-participant ratio

The older adult interviewees indicated that a team structure (e.g., a three-member team communicating with each other about roles and playing strategies) based on friendly competition would be appealing. Generally, the administrators recommended teams of eight to ten players per session and an instructor-to-participant ratio of 1:5.

## Strengths and limitations

This study has several strengths. First, to the best of our knowledge, this is the first study to apply the modified SM approach to the design of center-based eSports interventions for middle-aged and older adults in HK. Second, a large sample, 49 interviewees ranging widely in terms of age, membership to public and private community and elderly centers, and experience with eSports, was recruited; administrators with experience in PA programs were also interviewed. Such sampling representativeness may enhance the generalizability of this study result. Third, semistructured interviews facilitated in-depth discussion of the topics of interest. The interviews provided a data set that increased the applicability of our findings. This study also has some limitations. First, although the differences between eSports and exergaming was explained to the participants, they may not have fully grasped them, which could have affected their responses to our questions. Second, we adopted the five P’s instead of the seven P’s. More exploration about the remaining 2 Ps (i.e., physical evidence, and processes) is needed in the future. Third, our study’s result may provide a good reference to related studies overseas and so more exploratory study in this topic is warranted in other countries.

## Conclusion

This study applied the five P’s of an extended marketing mix to create and promote center-based eSports interventions that would encourage the participation of middle-aged and older adults. The involvement of administrators (some of whom also acted as program instructors) and participants is crucial to the interventions. We determined the applicability of a modified SM model for eSports to encourage PA among middle-aged and older adults and receive its health benefits. The intervention can motivate research on the design of successful interventions, convey the benefits of eSports and exergaming to older adults, and ensure healthy and active aging in HK in the long term. This study also revealed a need for further evidence-based research on the frequency and duration of interventions before definitive conclusions can be drawn regarding the use of eSports to improve health and quality of life for middle-aged and older adults.
